# P-1917. Hospital-Acquired Infections and Their Impact on Length of Stay for Veterans with COVID-19: A Nationwide Cohort Study

**DOI:** 10.1093/ofid/ofae631.2077

**Published:** 2025-01-29

**Authors:** Haojia Li, Karim Khader, Barbara E Jones, Michael Rubin, Yue Zhang

**Affiliations:** University of Utah, Salt Lake City, Utah; University of Utah, Salt Lake City, Utah; University of Utah and Salt Lake City VA Healthcare System, Salt Lake City, Utah; University of Utah, Salt Lake City, Utah; University of Utah, Salt Lake City, Utah

## Abstract

**Background:**

The COVID-19 pandemic has intensified concerns about hospital-acquired infections (HAI) due to multidrug-resistant organisms (MDRO) in hospitalized patients. This study assesses the excess length of stay (LOS) attributed to MDRO HAI across all acute Veterans Affairs (VA) hospitals.

**Methods:**

A retrospective cohort study was conducted within the VA healthcare system, including 115,000 hospital admissions from March 1, 2020, to August 31, 2022. We included 71,432 COVID-19 patients admitted within 14 days of a lab-confirmed diagnosis, excluding cases with community-acquired infections, non-MDRO HAIs, and readmissions. We utilized balanced risk-set matching (RSM) and mixed integer programming (MIP) to optimize covariate balance via Mahalanobis distance among key variables including demographic data, comorbidities, and both time-independent and -dependent treatments. Standardized mean differences (SMD) were used to ensure covariate balance, with an absolute value of less than 0.1 considered balanced.

**Results:**

We matched 1,088 patients who experienced HAI with MDRO. Covariate balance was achieved, with only minor deviations (SMD slightly over 0.1). The mixed-effect Poisson regression model adjusted for temporal and facility-level factors revealed that HAIs associated with MDRO resulted in a 32% longer LOS compared to the non-HAI group (95% CI: 30% - 35%; mean LOS: 26.8 days vs. 20.5 days; median LOS: 22 days vs. 16 days).

**Conclusion:**

This study highlights the significant impact of HAIs due to MDROs on the length of hospital stays among veterans with COVID-19. The findings underscore the need for enhanced infection control practices and targeted strategies to mitigate the burden of HAIs in this vulnerable population. Further research should explore the specific mechanisms by which HAIs extend hospitalization and the potential for intervention at various levels of healthcare delivery.

**Disclosures:**

All Authors: No reported disclosures

